# Brazilian propolis mitigates impaired glucose and lipid metabolism in experimental periodontitis in mice

**DOI:** 10.1186/s12906-016-1305-8

**Published:** 2016-08-30

**Authors:** Mayuka Nakajima, Kei Arimatsu, Takayoshi Minagawa, Yumi Matsuda, Keisuke Sato, Naoki Takahashi, Takako Nakajima, Kazuhisa Yamazaki

**Affiliations:** 1 Research Unit for Oral-Systemic Connection, Laboratory of Periodontology and Immunology, Division of Oral Science for Health Promotion, Niigata University Graduate School of Medical and Dental Sciences, 5274 Gakkocho 2-ban-cho, Chuo-ku, Niigata 951-8514 Japan; 2 Division of Periodontology, Department of Oral Biological Science, Niigata University Graduate School of Medical and Dental Sciences, Niigata, Japan; 3 Division of Dental Educational Research Development, Niigata University Graduate School of Medical and Dental Sciences, Niigata, Japan

**Keywords:** Periodontitis, Propolis, *Porphyromonas gingivalis*, Liver, Adipose tissue, Endotoxemia

## Abstract

**Background:**

Periodontitis has been implicated as a risk factor for metabolic disorders associated with insulin resistance. Recently, we have demonstrated that orally administered *Porphyromonas gingivalis*, a representative periodontopathic bacterium, induces endotoxemia via reduced gut barrier function coupled with changes in gut microbiota composition, resulting in systemic inflammation and insulin resistance. Propolis, a resinous substance collected by honeybees from leaf buds and cracks in the bark of various plants, can positively affect metabolic disorders in various experimental models. In this study, we thus aimed to clarify the effect of propolis on impaired glucose and lipid metabolism induced by *P. gingivalis* administration.

**Methods:**

Eight-week-old male C57BL/6 mice were orally administered *P. gingivalis* strain W83*,* propolis ethanol extract powder with *P. gingivalis,* or vehicle. We then analyzed the expression profile of glucose and lipid metabolism-related genes in the liver and adipose tissues. Serum endotoxin levels were also evaluated by a limulus amebocyte lysate test. In addition, we performed histological analysis of the liver and quantified alveolar bone loss by measuring the root surface area on the lower first molar.

**Results:**

Oral administration of *P. gingivalis* induced downregulation of genes that improve insulin sensitivity in adipose tissue (*C1qtnf9*, *Irs1*, and *Sirt1*), but upregulation of genes associated with lipid droplet formation and gluconeogenesis (*Plin2*, *Acox*, and *G6pc*). However, concomitant administration of propolis abrogated these adverse effects of *P. gingivalis*. Consistent with gene expression, histological analysis showed that administered propolis suppressed hepatic steatosis induced by *P. gingivalis*. Furthermore, propolis inhibited the elevation of serum endotoxin levels induced by *P. gingivalis* administration. Contrary to the systemic effects, propolis had no beneficial effect on alveolar bone loss.

**Conclusion:**

These results suggest that administration of propolis may be effective in suppressing periodontopathic bacteria-induced metabolic changes that increase the risk of various systemic diseases.

## Background

Periodontal diseases are mainly chronic infectious diseases resulting from responses to a complex dental plaque microbiome containing various periodontopathic bacteria species. Epidemiological studies suggest that periodontitis is a risk factor for various systemic diseases, such as type 2 diabetes [1, 2], atherosclerotic vascular diseases [3, 4], and non-alcoholic fatty liver disease [5]. Among the various periodontopathic bacteria, considerable research has been focused on the role of *Porphyromonas gingivalis* as a possible mechanism linking periodontal and other human diseases, due to its unique pathogenicity [6] and its association with various diseases. Although the possible significance of common susceptibility cannot be discounted, there are several hypothetical causal mechanisms linking periodontal disease and systemic diseases. First, bacteria from dental plaque invade gingival tissue through ulcerated sulcular epithelial linings of periodontal pockets and then disseminate into systemic circulation. Second, various proinflammatory cytokines produced in inflamed periodontal tissue, which can also enter systemic circulation, are delivered to various tissues and organs and thereby induce an inflammatory response [7].

Interestingly, links between the diseases related to periodontitis and dysbiosis of the gut microbiota are becoming more evident [8, 9]. Recently, we revealed that oral administration of *P. gingivalis* altered gut microbiota and elicited endotoxemia, thereby inducing systemic inflammation and insulin resistance [10, 11].

Propolis is a plant product collected by honeybees as a resinous mixture from various plants that is mixed with beeswax and other bee secretions. Although the chemical composition of propolis depends on its location of origin, it basically contains beneficial substances, such as phenolic acids, flavonoids, and vitamins [12, 13]. However, the details of the main ingredients have not yet been disclosed.

Propolis has been used as a folk medicine since ancient times due to its anti-inflammatory [14, 15], anti-microbial [16, 17], anti-oxidant [12], and anti-tumour properties [18, 19]. In addition, previous studies have shown the beneficial effect of propolis on diabetes mellitus. Fuliang et al. revealed that administering propolis improved blood glucose levels and modulates glucose and blood lipid metabolism in experimental rat models of diabetes [20]. Kitamura et al. demonstrated that the propolis extract restored glucose intolerance and insulin resistance, as well as blood glucose and plasma cholesterol levels using *ob*/*ob* mice [21]. Furthermore, because of its effectiveness on enterobacteria [22], propolis is expected to replace probiotics as a novel regulator of gut microbiota.

In the present study, we evaluated whether systemic inflammation and insulin resistance induced by periodontopathic bacteria through intestinal reactions could be suppressed by administering propolis.

## Methods

### Mice

Eight-week-old male C57BL/6 mice were obtained from Japan SLC, Inc. (Shizuoka, Japan). The mice were acclimatized under specific pathogen-free conditions and fed regular chow and sterile water until the commencement of infection at nine weeks of age.

### Bacterial cultures

*P. gingivalis* (strain W83) was cultured in modified Gifu anaerobic medium (GAM) broth (Nissui, Tokyo, Japan) in an anaerobic jar (Becton Dickinson Microbiology System, Cockeysville, MD, USA) in the presence of an AnaeroPack™ (Mitsubishi Gas Chemical Co. Inc., Tokyo, Japan) for 48 h at 37 °C. Bacterial suspensions were prepared in phosphate-buffered saline (PBS) without Mg^2+^/Ca^2+^ using established growth curves and spectrophotometric analysis. The number of colony-forming units (CFUs) was standardized by measuring optical density at 600 nm.

### Oral administration

The murine experimental periodontitis model was developed according to Baker et al. with slight modifications [23]. In the present study, mice did not receive antibiotic pretreatment before *P. gingivalis* administration since systemic antimicrobial treatment affects the gut microbiota composition and alters metabolism [24]. In addition, we have observed *P. gingivalis*-induced alveolar bone resorption without antibiotic pretreatment [25]. A total of 10^9^ CFUs of live *P. gingivalis* suspended in 100 μL PBS with 2 % carboxymethyl cellulose (Sigma-Aldrich, St. Louis, MO, USA) was given to each mouse via a feeding needle three times a week for 5 weeks. The number of administered bacteria was determined based on the number of *P. gingivalis* contained in saliva of patients with severe periodontitis [26] and adjusted for body weight. The control group was sham-administered 100 μL PBS with 2 % carboxymethyl cellulose without *P. gingivalis*. In the propolis group, powdered ethanol extracts of propolis mixed with 2 % carboxymethyl cellulose (200 mg/kg weight) were administered every day during the experimental period in addition to *P. gingivalis* administration three times a week for 5 weeks. The propolis ethanol extract, including 55 % propolis extract as solid content, standardized to contain minimum 8.0 % artepillin C, was obtained from Yamada Bee Company, Inc. (Okayama, Japan).

During the experimental period, all mice were allowed to eat and drink *ad libitum*. One day after the final treatment, mice were euthanized with CO_2_ and their tissues were collected.

### Analysis of gene expression in the liver and adipose tissues

Total RNA from the liver and adipose tissues were extracted using TRI Reagent® (Molecular Research Center, Inc., Cincinnati, OH, USA). cDNA was synthesized with Transcriptor Universal cDNA Master (Roche Molecular Systems, Inc., Branchburg, NJ, USA). Primers and probes specific for real-time PCR were purchased from Life Technologies Corporation. Reactions were carried out in a final volume of 25 μL in a LightCycler® 96 System (Roche) using TaqMan Gene Expression Assays (Life Technologies Corporation) containing 900 nM each of the forward and reverse primers and a 250 nM probe. The reactions consisted of a 10-min incubation at 95 °C, followed by 45 cycles of a 2-step amplification procedure, consisting of annealing/extension at 60 °C for 1 min and denaturation for 15 s at 95 °C. LightCycler® 96 software (Roche) was used to analyze the standards and carry out the quantification. The relative quantity of each mRNA was normalized to the relative quantity of glyceraldehyde-3-phosphate dehydrogenase (GAPDH) mRNA.

### Quantification of alveolar bone loss

The amount of bone loss was assessed from images obtained using a stereomicroscope fitted with a video image marker measurement system (DP2-BSW; Olympus, Tokyo, Japan). We determined the area surrounded by the margin of the submaxillary alveolar bone crest and the cement-enamel junction on the lingual side of the first molar. We measured alveolar bone loss in the mice in a blind manner.

### Endotoxin assay

Endotoxin levels were determined in sera collected at the end of the experimental period using a Limulus amebocyte lysate test (QCL-1000TM, BioWhittaker, Walkersville, MD, USA) according to the manufacturer’s instructions. Serum samples were diluted 1-to-4 for the assay. Optical densities were measured using an ELISA plate reader (Model 680, Bio-Rad Laboratories, Hercules, CA, USA) at 405 nm.

### Histological analysis of the liver tissue

The liver tissues of three mice from each group were fixed in 10 % formalin. Briefly, samples were embedded in paraffin, sectioned, and stained with hematoxylin and eosin.

### Statistical analysis

Prior to beginning the present study, the sample size calculation was performed based on the data from our previous study. With an alpha error of 0.05 and a statistical power of 0.8, the sample size was calculated to require more than seven animals in each group. This was verified by post-hoc analyses. The data were tested for normality using the Kolmogorov-Smirnov test. Because some, but not all, data sets showed a parametric distribution even in a particular gene expression among different treatment groups, all data were assessed by the Kruskal-Wallis test with a post-hoc Dunn’s multiple comparison test using GraphPad Prism® (GraphPad Software, Inc., La Jolla, CA). A probability value of *p* < 0.05 was considered statistically significant.

## Results

### Effect of *P. gingivalis* administration and/or propolis on the body weight

**Fig. 1:**
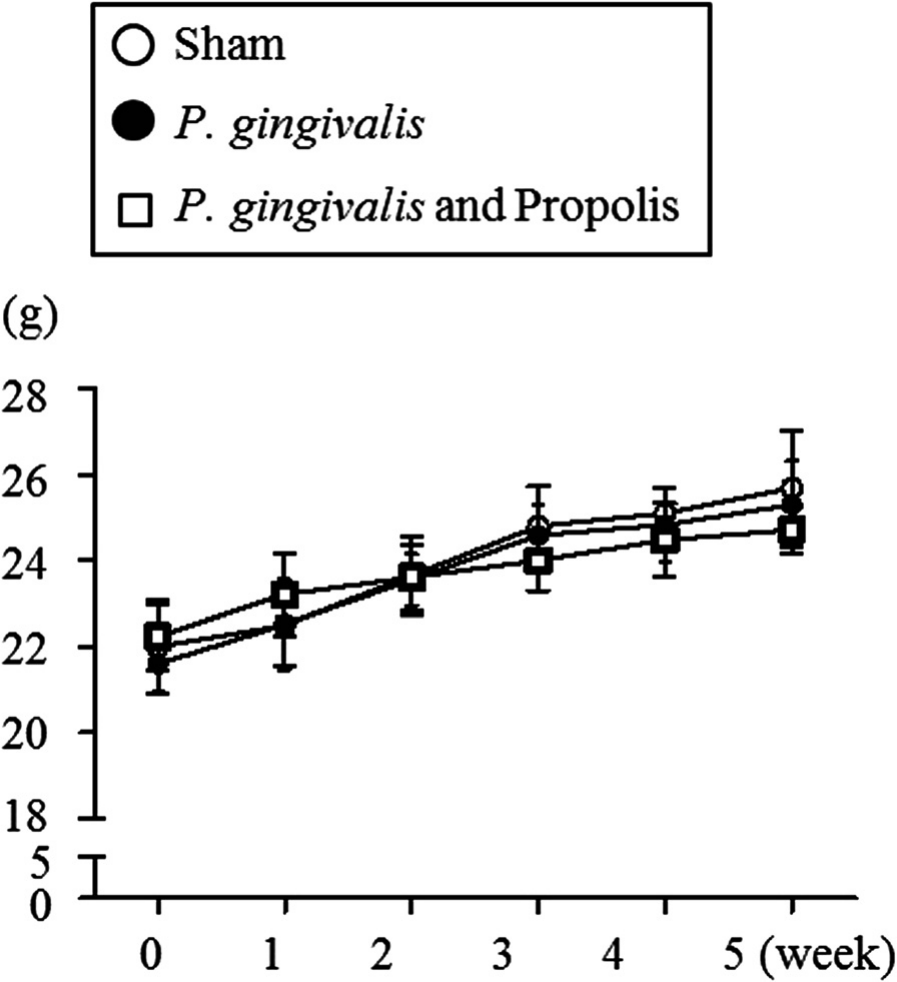
Effect of oral administration of *P. gingivalis* or *P. gingivalis* and propolis on body weight. Body weight changes during experimental period in Sham-administered mice (*N* = 7), *P. gingivalis*-administered mice (*N* = 7), and *P. gingivalis* and propolis-administered mice (*N* = 8). All data are mean ± SD. (*p < 0.05, Kruskal-Wallis test with post-hoc Dunn’s multiple comparison test)

After treatment with *P. gingivalis* and propolis, there were no differences among the sham-administered, *P. gingivalis*-administered, or *P. gingivalis* and propolis-administered groups in body weight (Fig. 1) or food intake.

### Effect of propolis on the altered gene expression in the liver by *P. gingivalis* administration

**Fig. 2:**
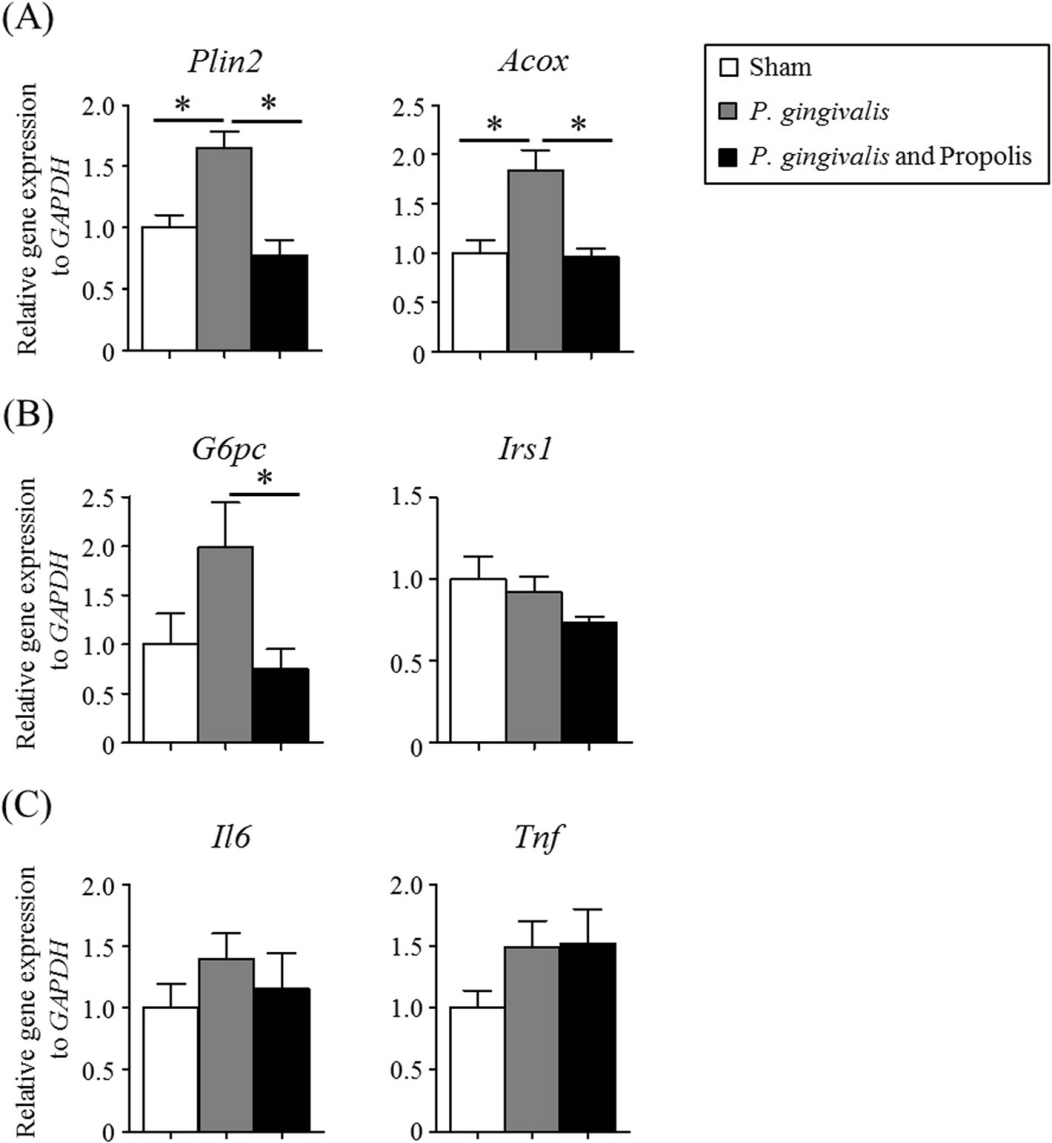
Effect of oral administration of *P. gingivalis* or propolis on gene expression in the liver. Expression of genes related to lipid metabolism **a**, glucose metabolism **b**, and inflammation **c**. Sham-administered mice (*N* = 7), P. gingivalis-administered mice (*N* = 7), Propolis-administered mice (*N* = 8). The relative mRNA expressions of the genes of interest were normalized to the relative quantity of glyceraldehyde-3-phosphate dehydrogenase (GAPDH) mRNA. All data are means ± SD. (*p < 0.05, Kruskal-Wallis test with post-hoc Dunn’s multiple comparison test)

Oral administration of *P. gingivalis* significantly increased gene expression of *Plin2* and *Acox*, both of which are associated with lipid metabolism in the liver, while these gene expressions were markedly reduced in the propolis-administered mice (Fig. 2a). Furthermore, administration of propolis suppressed *G6pc* expression, which positively regulates gluconeogenesis, compared to mice administered *P. gingivalis* alone. Conversely, there was no significant change in the expression of the insulin signaling gene *Irs1* among the three groups (Fig. 2b). Although gene expression of the proinflammatory cytokines IL-6 and TNF-α tended to be elevated from the administered *P. gingivalis*, administration of propolis had no effect on the expression of these genes (Fig. 2c).

**Fig. 3:**
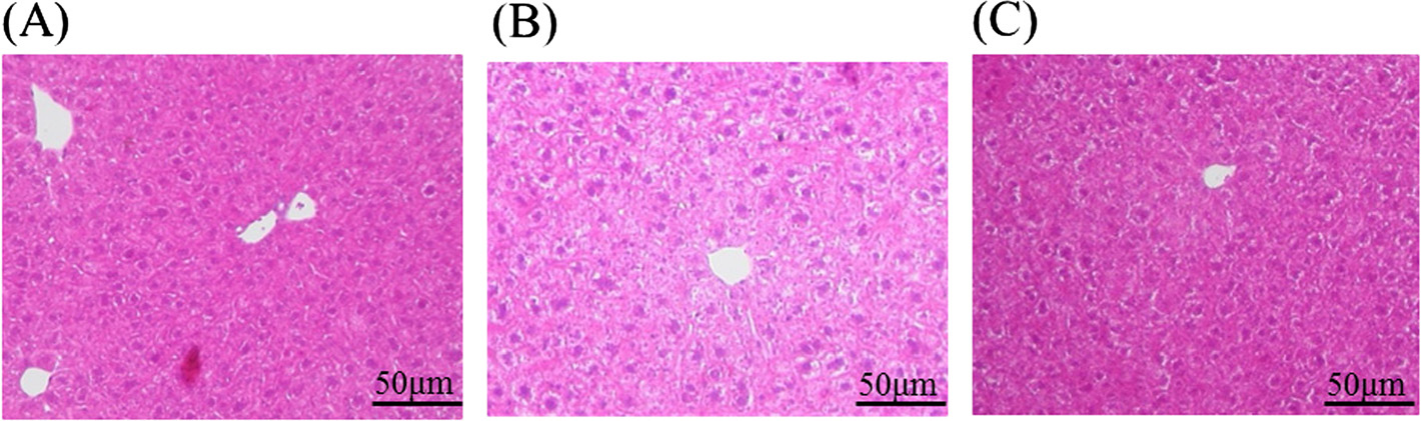
Histological analysis of the liver tissue. Sections of liver tissue in **a** Sham-administered mice, **b** *P. gingivalis*- administered mice, and **c** *P. gingivalis* and propolis-administered mice were H-E stained. Representative results are shown (*N* = 3 in each group)

Administering propolis led to decreased *Plin2* and *Acox* expression, which were associated with the mitigation of hepatic steatosis induced by *P. gingivalis* administration (Fig. 3).

### Effect of propolis on the altered gene expression in the adipose tissue by *P. gingivalis* administration

**Fig. 4:**
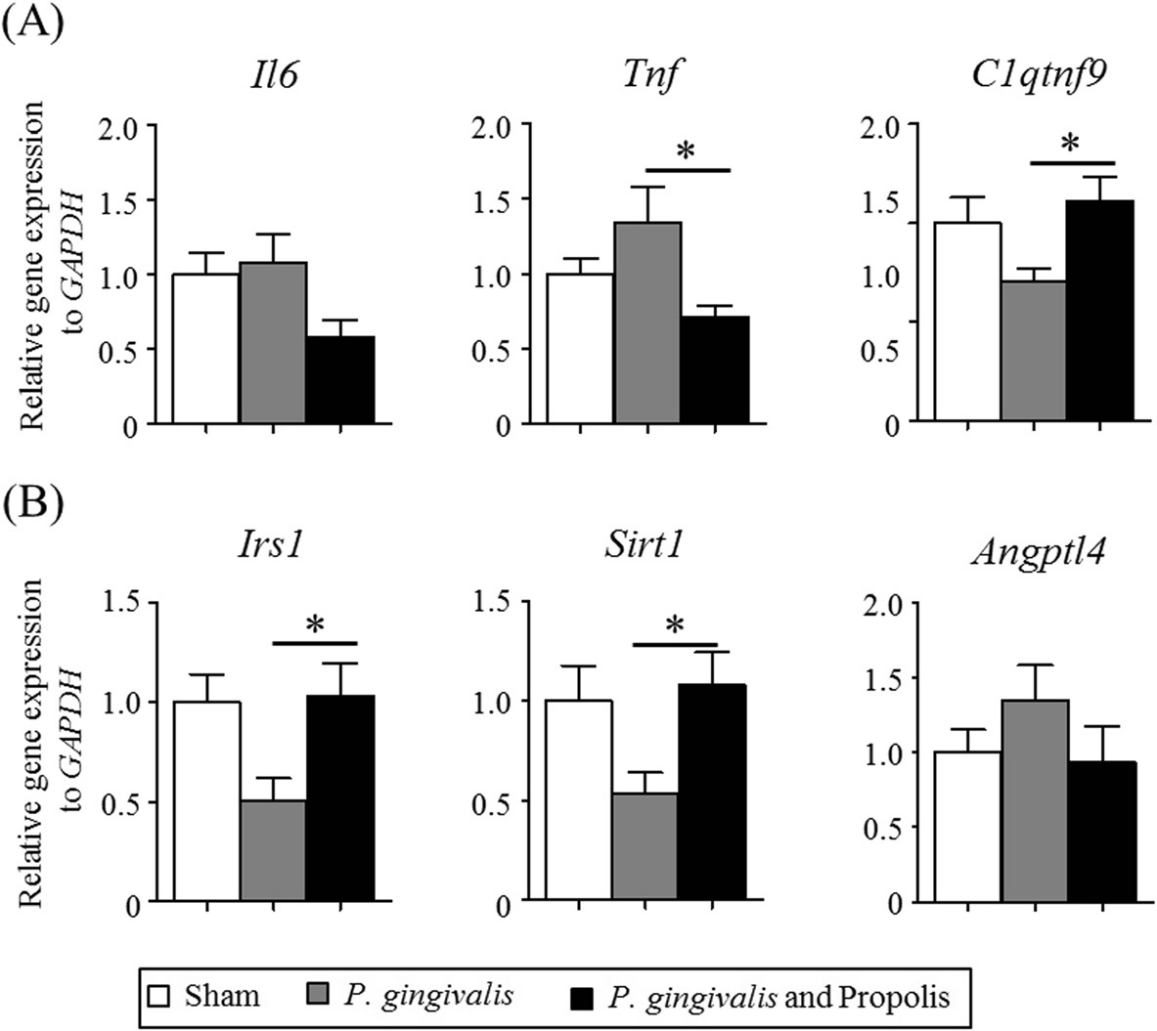
Effect of oral administration of *P. gingivalis* or propolis on gene expression in the epididymal adipose tissue. **a** Relative gene expression of adipocytokine. **b** Relative expression of genes related to glucose metabolism. Sham-administered mice (*N* = 7), P. gingivalis-administered mice (*N* = 7), P. gingivalis and propolis-administered mice (*N* = 8). The relative mRNA expressions of the genes of interest were normalized to the relative quantity of glyceraldehyde-3-phosphate dehydrogenase (GAPDH) mRNA. All data are means ± SD. (*p < 0.05, Kruskal-Wallis test with post-hoc Dunn’s multiple comparison test)

*Il6*, *Tnf*, *C1qtnf9*, and *Adipoq* are known as adipocytokines that are expressed in adipocytes to modulate glucose and lipid metabolism. The gene expression of the proinflammatory cytokines IL-6 and TNF-α, which suppress insulin signals, tended to be higher in the *P. gingivalis*-administered mice. Conversely, *C1qtnf9*, a gene that improves insulin sensitivity, was more frequently downregulated in the *P. gingivalis*-administered mice. Administration of propolis ameliorated these changes to gene expression induced by *P. gingivalis* administration (Fig. 4a). Furthermore, the genes that improve insulin sensitivity, *Irs1* and *Sirt1*, were downregulated in *P. gingivalis*-administered mice. However, propolis administration reversed the effect of *P. gingivalis* administration on the expression of these genes. In addition, administration of propolis suppressed the *P. gingivalis*-induced increased expression of *Angptl4*, a gene that is supposed to increase insulin resistance (Fig. 4b).

### Effect of propolis on the serum endotoxin levels in *P. gingivalis*-administered mice

**Fig. 5:**
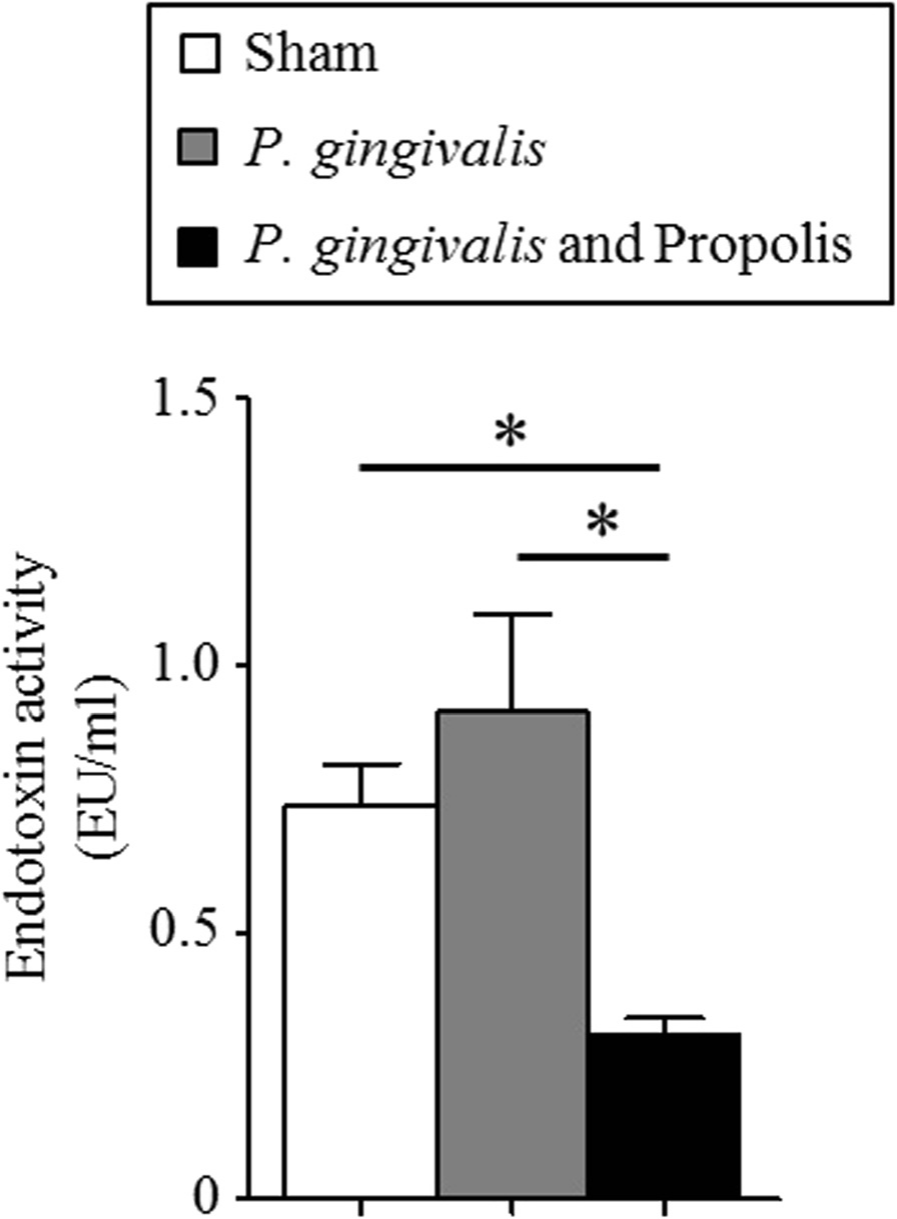
Effect of oral administration of *P. gingivalis* or propolis on endotoxemia. Serum endotoxin (LPS) concentration (EU/mL) were evaluated in Sham-administered mice (*N* = 7), *P. gingivalis*-administered mice (*N* = 7), and *P. gingivalis* and propolis-administered mice (*N* = 8). All data are means ± SD. (*p < 0.05, Kruskal-Wallis test with post-hoc Dunn’s multiple comparison test)

Oral administration of *P. gingivalis* increased serum endotoxin levels, but was not statistically significant. Administration of propolis not only repressed this increase, but also further lowered serum endotoxin levels below those of untreated mice (Fig. 5).

### Effect of propolis on the alveolar bone resorption in *P. gingivalis*-administered mice

**Fig. 6:**
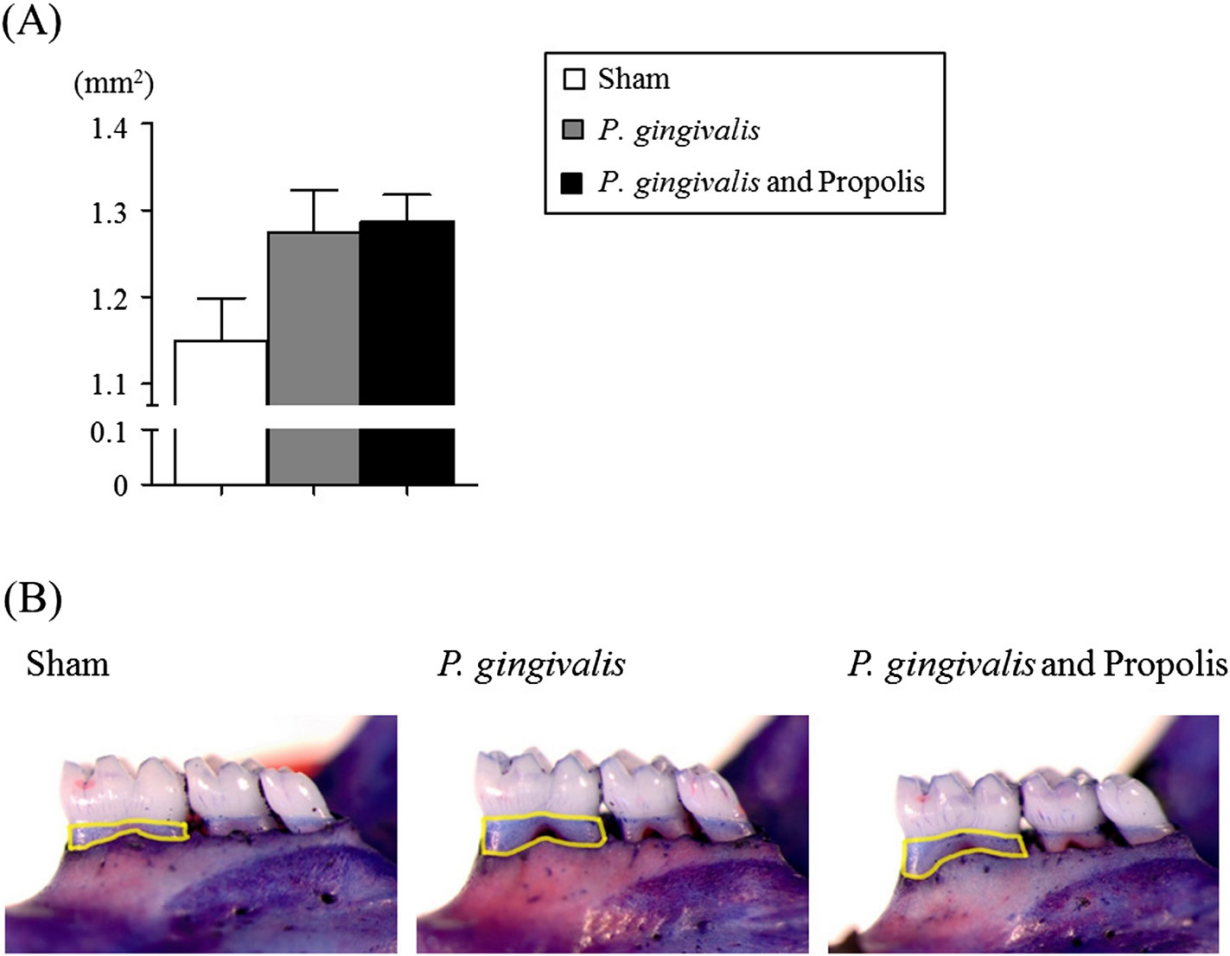
Quantification of alveolar bone loss. **a** The area surrounded by the margin of the submaxillary alveolar bone crest and the cement-enamel junction on the lingual side of the first molar was determined using a stereoscopic microscope. The image analysis was carried out in Sham-administered mice (*N* = 7), *P. gingivalis*-administered mice (*N* = 7), and *P. gingivalis* and propolis-administered mice (*N* = 8). All data are means ± SD. No significant difference was observed. **b** Photographs were taken after the removal of soft tissues. The measured area is displayed with a yellow line. Representative results are shown (*N* = 7 in Sham- and *P. gingivalis*-administered mice, *N* = 8 in *P. gingivalis* and propolis- administered mice)

As with the previous studies, oral administration of *P. gingivalis* induced significant alveolar bone resorption. In spite of the beneficial systemic effect of administering propolis, no effect was observed on the *P. gingivalis*-induced alveolar bone resorption (Fig. 6).

## Discussion

Periodontal disease is a chronic inflammatory disease likely resulting from dysbiosis of the oral microbiota. Epidemiological studies indicate its association with an increased risk of various diseases, such as diabetes [1, 2], atherosclerotic vascular diseases [3, 4], and rheumatoid arthritis [5]. The underlying mechanisms linking periodontal disease and these diseases have been considered to be endotoxemia and proinflammatory cytokines derived from gingival lesions [7]. We have shown that repeated oral inoculation of *P. gingivalis* induced elevation of serum inflammatory markers (serum amyloid A and IL-6) in mice [27]. However, in this mouse model, administered *P. gingivalis* was not detected in the blood or insulin target tissues and there was little inflammation in the gingival tissues, suggesting that conventional mechanisms for the relationship between periodontitis and systemic diseases may not be applicable, at least in this mouse model. Recently, we have also demonstrated altered gut bacterial composition by repeated oral administration of *P. gingivalis* as a novel underlying mechanism [10, 11]. Since the gut microbial changes are associated with decreased gut barrier function, as evidenced by reduced expression of the tight junction protein gene (*Tjp1*) and elevated endotoxin levels in the systemic circulation in our previous studies [10, 11], inflammation of adipose and liver tissue seen in the present study was expected to be from bacteria influx and/or bacterial products into these tissues.

In the present study, we have demonstrated that oral administration of *P. gingivalis* altered gene expression associated with tissue-specific pathological changes relating to metabolic syndromes. In the liver, the expression of *Plin2* and *Acox1* was significantly upregulated. *Plin2* is reported to be strongly associated with lipid droplet formation in the liver [28] and *Acox1* is involved in fatty acid oxidation [29]. Both genes play important roles in non-alcoholic fatty liver diseases. *P. gingivalis* administration also seemed to affect glucose metabolism by increasing the expression of *G6pc. G6pc* positively regulates gluconeogenesis and can increase blood glucose levels [30].

Adipose tissue plays an important role in mediating insulin sensitivity through various molecules, including adipocytokines. *C1qtnf9* encoding C1q/TNF-related protein (CTRP)9 is a novel and highly conserved paralog of adiponectin and has salutary effects on glucose metabolism and vascular function [31, 32]. *Irs1* [33] and *Sirt1* contribute to improving insulin sensitivity. SIRT1 not only stimulates a glucose-dependent insulin secretion from pancreatic beta cells, but also directly stimulates insulin signaling pathways in insulin-sensitive organs [34]. *Tnf* and *Il6* are further adipocytokines with strong, natural proinflammatory traits that negatively impact insulin signaling. *Angptl4* can also be involved in insulin resistance [35]. Taken together, oral administration of *P. gingivalis* has harmful effects on insulin sensitivity.

Administration of propolis during the experimental period seemed to show no serious adverse effects, as indicated by no change in body weight among the groups. In support of previous studies [21, 36], administering propolis alleviated the detrimental effects on glucose and lipid metabolism induced by oral administration of *P. gingivalis*. Although we have not analyzed the detailed mechanisms by which propolis suppress these harmful effects, it is possible that administration of propolis could affect gut barrier function, either by alteration of gut microbiota composition or direct elevation of gut barrier function. In support of this idea, our previous studies clearly demonstrate that oral administration of *P. gingivalis* changes gut microbiota composition, inducing sustained endotoxemia and systemic inflammation [10, 11]. Another previous work shows high-fat diet-induced obesity influences gut microbiota and induces metabolic endotoxemia [37]. This endotoxemia is considered to be a result of reduced gut barrier function. In the present study, we also observed that administering *P. gingivalis* tended to increase serum endotoxin levels, while administering propolis completely abrogated the effect of *P. gingivalis.* Although administering propolis had no effect on alveolar bone resorption induced by *P. gingivalis* indicating no local effects on periodontal tissue, low levels of endotoxin activity in the propolis-administered mice could be mediated by anti-microbial effect on *P. gingivalis*. In this regard, several studies have demonstrated a significant antimicrobial effect of propolis on periodontopathic bacteria such as *P. gingivalis* [38–41] and an inhibitory effect on alveolar bone resorption in ligature-induced periodontitis in rats [42, 43]. However, our study demonstrated no suppressive effect of propolis on alveolar bone resorption. The difference between our study and the study by Toker et al. [43] could be due at least in part to the different methods for inducing experimental periodontitis. Alternatively, propolis administration may have induced changes in gut microbiota composition or strengthened the gut barrier function. One previous study showed that propolis upregulated tight junction proteins in Caco-3 cells and that *in vivo* administration of propolis increased colonic epithelial ZO-1 expression [44]. Furthermore, it has been demonstrated that a gut metabolite of linoleic acid ameliorates inflammation-induced intestinal epithelial barrier impairments, suggesting that gut microbiota composition is crucial for the maintenance of gut barrier function [45]. Thus, it is reasonable to consider the beneficial effects of propolis on gut microbiota.

## Conclusion

Although further studies are needed to clarify the underlying mechanism by which propolis suppresses *P. gingivalis*-induced metabolic disturbance, our results suggest that administration of propolis may be effective in suppressing periodontopathic bacteria-induced metabolic changes that increase the risk of various systemic diseases.
